# Prevalence and characteristics of dental and periodontal disease in Western European hedgehogs (*Erinaceus europaeus*) admitted into an animal shelter in northwestern Germany

**DOI:** 10.1007/s11259-026-11287-0

**Published:** 2026-05-22

**Authors:** Ines Stegmaier-Länge, Gidona Goodman, Lian Thomas

**Affiliations:** 1 Royal (Dick) School of Veterinary Studies, University of Edinburgh, Edinburgh, United Kingdom; 2 Bremer Tierschutzverein e.V., Bremen, Germany; 3 International Livestock Research Institute, Nairobi, Kenya

**Keywords:** Western European hedgehog, *Erinaceus europaeus*, Dental disease, Periodontal disease, Calculus, Gingivitis

## Abstract

Dental and periodontal disease are common findings in Western European hedgehogs (*Erinaceus europaeus*) admitted into rehabilitation but are rarely studied. This study aimed to collect data on the prevalence and characteristics of dental and periodontal disease in Western European hedgehogs admitted into an animal shelter in northwestern Germany. A full body examination including a dental assessment was conducted on all 95 hedgehogs admitted between 15 August 2024 and 15 April 2025. Indices judging calculus, gingivitis, gingival recession, and tooth mobility were assessed and added up to an overall dental examination index representing the severity of the disease. Disease prevalence and risk factors and clinical signs as potential predictive factors for the presence of the disease were examined. The prevalence among the overall study population was 44.2% (95% CI [34.7–54.7]). Calculus was found as the most common pathology. For gingival recession and the overall examination index, low and high scores were more common than medium scores. In the final multivariate model, a higher body weight was a positive predictive factor for the presence of dental/periodontal disease among both juvenile hedgehogs and those past their first calendar year (*p* < 0.001; OR = 1.011). Further research is needed to understand the risk factors for the disease and to assess the significance of dental and periodontal disease on individual hedgehogs and the declining overall population.

**Supplementary Information:**

The online version contains supplementary material available at 10.1007/s11259-026-11287-0.

## Introduction

The hedgehog family is generally prone to dental and periodontal disease (Poduschka and Poduschka 1986; Simone-Freilicher and Hoefer 2004; Heatley 2009; Chaprazov et al. 2014). A prevalence of 0.75 to 8.4% has been described for wild hedgehogs examined in post-mortem examinations or during rehabilitation (Zuhrt 1958; Doepke 2002; Koegel 2009; Molina-Lopez et al. 2024).

Periodontal disease is the progressive attachment loss of a tooth (Stepaniuk 2019). The initial, reversible stage of periodontal disease is gingivitis, where inflammation caused by the bacteria in plaque is restricted to the gingiva, showing as erythema, oedema, and bleeding. Periodontitis, the later, active stage of periodontal disease, is an inflammation of the periodontal ligament and the alveolar bone which can become visible as gingival recession (Niemiec 2008; Stepaniuk 2019). The final stage of periodontal disease is tooth loss, although spreading infection and inflammatory response can ultimately have other local or systemic consequences like a tooth-root abscess, osteomyelitis, and septicaemia (Niemiec 2008).

The causes for the development of dental and periodontal disease are poorly understood and suspected to be multifactorial (Stepaniuk 2019). An advanced age, male sex, and a soft diet are discussed as risk factors in hedgehogs (Sainsbury et al. 1996; Chaprazov et al. 2014; Bexton 2016). Weight loss and lethargy are mentioned as nonspecific symptoms, but no studies have been performed on clinical signs in Western European hedgehogs (Chaprazov et al. 2014; Bexton 2016). For different species, nasal and ocular discharge were shown to result from severe periodontal disease (Boulton 1985; Verstraete 2003; Niemiec 2008).

Rehabilitation centres in Germany and Europe report dental disease as a common, potentially increasing health problem among admitted hedgehogs. The current scientific literature does not confirm this subjective feeling. Furthermore, it rarely differentiates between the pathologies and does not characterise the severity of the disease. It is therefore difficult to draw conclusions on the true extent of these pathologies and their significance to the hedgehogs. This study aimed to collect information on the prevalence and characteristics, including predictive factors, of dental and periodontal disease in Western European hedgehogs admitted into rehabilitation in northwestern Germany.

## Materials and methods

### Data collection

All Western European hedgehogs admitted to Bremer Tierschutzverein e.V., an urban animal shelter in northwestern Germany, between 15 August 2024 and 15 April 2025 were included in the study. As part of the shelter’s intake protocol, a health check was performed for each hedgehog immediately upon arrival and a full veterinary examination, including the assessment of the dental status, was conducted under general anaesthesia once the hedgehog was stable. For five hedgehogs weighing 100 g or less, this was fully possible without anaesthesia. Each hedgehog’s weight, body length, body condition, age group, sex, abnormal findings for all body systems, and dental status were assessed. The hedgehogs’ age was grouped into juveniles within their first calendar year and hedgehogs older than their first calendar year with full adult dentition. This was assessed according to the time of the year of admittance and the morphological features body weight, body condition, body length, and dentition.

**Fig. 1:**
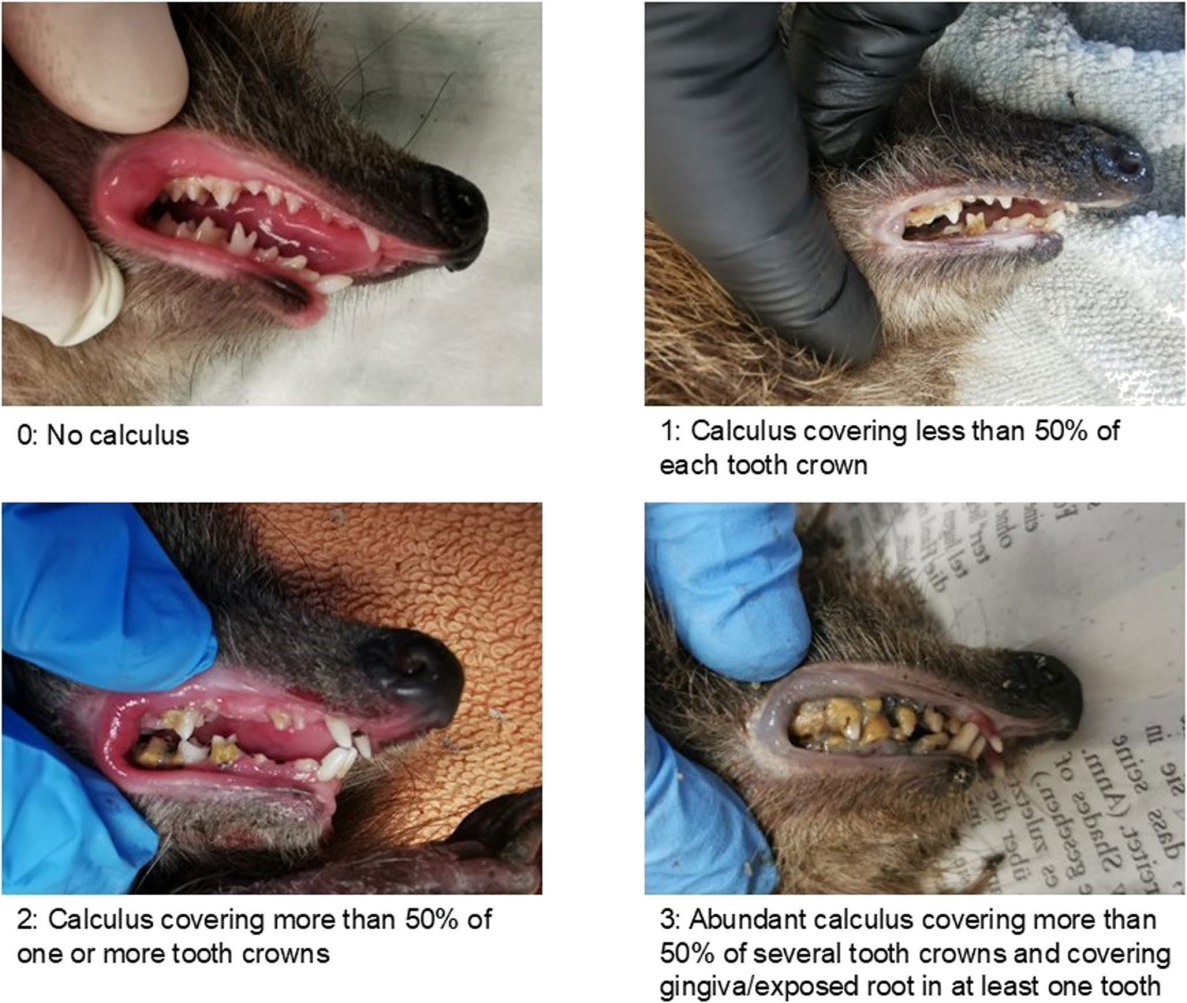
Calculus index (Photos: Ines Stegmaier-Länge)

During the dental examination, the following indices were assessed (a visual presentation of all indices and the used protocol are available in Online Resource 1):

Calculus index: See Fig. 1.

Gingival index:

- 0 Normal gingiva.
- 1 Mild gingivitis (redness and/or swelling, no bleeding).
- 2 Moderate gingivitis (redness, swelling, mild bleeding on palpation).
- 3 Severe gingivitis (redness, swelling, ulcerations and/or spontaneous bleeding).

Periodontal index:

- 0 No gingival recession.
- 1 Gingival recession in one or more teeth, furcation exposed in one multirooted tooth maximum.
- 2 Furcation exposed in more than one multirooted tooth, periodontal probe does not reach through the furcation in any teeth.
- 3 Furcation exposed in more than one multirooted tooth, periodontal probe reaches through the furcation in one or more teeth.

Mobility index:

- 0 No noticeable mobility.
- 1 Mildly increased movement of one or more teeth that stay stable within the alveolus.
- 2 Up to four teeth loose and likely to fall out soon, no or mildly increased movement in other teeth.
- 3 More than four teeth loose and likely to fall out soon, no or mildly increased movement in other teeth.

### Data management and analysis

Dental/periodontal disease was considered present as soon as one of the indices exceeded zero. An overall dental examination index was calculated by adding up the four indices. The indices were developed following the examinations in raccoons by Hungerford et al. (1999), the examinations in boar by Malmsten et al. (2015), and the indices for veterinary dentistry in cats and dogs described by Stepaniuk (2019), taking into consideration the pathologies relevant for hedgehogs (Chaprazov et al. 2014). For ten hedgehogs dying in captivity no gingival index and no dental examination index were attributed.

Statistical data analysis was performed using IBM SPSS Statistics for Windows, version 29.0. The (8-month period) prevalence of dental/periodontal disease and the 95% confidence interval were determined. Bivariate analysis was performed to examine for an association between the presence or absence of the disease and possible risk factors and clinical sign as potential predictive factors. For nominal predictors, a Chi-square test was performed when cell counts were above or equal to five for each cell, and a Fisher’s exact test was performed if a cell count was lower than 5. For continuous data, the Student’s t-test for independent samples was used in case of a normal distribution and the Mann-Whitney U test was used if data did not follow a normal distribution. Predictors with a *p*-value below 0.1 were included in a logistic regression model. Body length was not included in the model to avoid multicollinearity with body weight. The final adjusted model was fitted after a backward stepwise selection of variables following the likelihood ratio criterion.

## Results

A total of 95 hedgehogs were admitted for rehabilitation during the study period and were therefore included in the study. The overall prevalence of dental and/or periodontal disease in the study population was 44.2% (95% CI [34.7–54.7]).

**Fig. 2:**
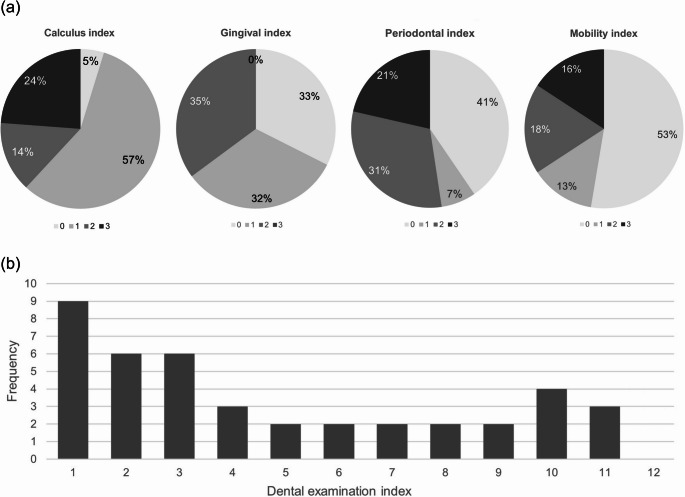
Frequency of (**a**) the four dental indices investigated among all hedgehogs with dental and/or periodontal disease upon arrival, 0 meaning no pathology and 3 being the highest possible score; and (**b**) the dental examination index among hedgehogs with dental and/or periodontal disease, calculated by adding up the four individual indices for each hedgehog

Figure 2 presents the results of the dental assessment in hedgehogs with dental/periodontal disease. Calculus was found as the most common pathology. Almost every hedgehog with dental/periodontal disease had calculus, with index 1 being the most common one. Gingival indices 0, 1, and 2 were evenly distributed whereas gingival index 3 was not found in any hedgehog. Periodontal index 0 was the most common one, but once gingival recession was visible, most hedgehogs showed stage 2 or 3. Mobility of teeth was found as the least common pathology.

For the overall dental examination index representing the severity of the disease, the most common values were low with an additional peak at the upper end of the scale. The median dental examination index among all hedgehogs showing dental/periodontal disease was 3 (IQR 7). Pathologies not covered by the indices that were found in a small number of individuals were broken teeth (*n* = 3) and tooth root abscesses (*n* = 3).

The risk factors and clinical signs investigated as potential predictive factors for the presence of dental/periodontal disease are presented in Table 1.

**Table 1:** Investigated potential predictive factors for dental/periodontal disease in hedgehogs upon admittance into rehabilitation among the overall study population, and a bivariate comparison between hedgehogs with and without disease

Predictive factor	Overall study population	No disease	Dental/periodontal disease	
	*n* = 95/94^a^	*n* = 53/52^a^	*n* = 42	
	PR (95% CI) / *Mdn (IQR)	PR (95% CI) / *Mdn (IQR)	PR (95% CI)/ *Mdn (IQR)	*p*-value
Age > First calendar year (%)	40.0 (29.5–50.5)	7.5 (1.8–15.8)	81.0 (68-92.7)	< 0.001
Male sex (%)	45.3 (36.2–56.4)	50 (35.6–64.0)	40.5 (25.6–55.5)	0.409
*Body length (cm)	17.9 ± 7.4	14.4 ± 4.3	21.5 ± 4.0	< 0.001
*Body weight (g)	315 ± 406	171 ± 133	580 ± 284	< 0.001
Emaciation (%)	76.8 (68.4–85.3)	79.2 (67.7–89.6)	73.8 (60.4–87.0)	0.533
Nasal discharge/ sneezing/breathing noise (%)	11.6 (6.3–18.9)	5.7 (0.0-12.9)	19.0 (7.9–31.4)	0.056
Ocular discharge/swelling (%)	10.5 (5.3–16.8)	1.9 (0.0-6.1)	21.4 (10.0-34.2)	0.002

^a^sample size smaller for sex; CI: confidence interval; IQR: Interquartile Range; Mdn: Median; PR: prevalence

In the logistic regression analysis for predictive factors, only the age group and the body weight were kept in the final model after stepwise backward selection, with the age group being a confounder. The body weight was a significant positive predictor for the presence of dental/periodontal disease when the age group was held constant (*p* < 0.001, OR = 1.011, CI 95% [1.005–1.017]).

## Discussion

The disease prevalence of 44.2% found in this study is significantly higher than described in previous literature. While it is difficult to make direct comparisons due to methodological differences, two theories emerge on why this may be. Firstly, the differing methods of dental examination, and secondly, a potential increase in periodontal disease over time. Regarding the method of dental examination, both Koegel (2009) and Molina-Lopez et al. (2024) investigated hedgehogs’ medical records from rescue centres, part of which were presumably examined without anaesthesia. Therefore, the disease prevalence is likely underestimated in these studies. Supporting the theory of an increase in prevalence over time, Zuhrt (1958) and Doepke (2002) found a lower prevalence of calculus in dead hedgehogs previously admitted into rehabilitation centres and veterinary clinics or found dead in the wild, respectively. As post-mortem examinations were carried out, it can be assumed that prevalence would not have been underestimated in these studies. As they focused on hedgehogs who were rescued or found dead, their study population may be considered comparable to ours.

A limitation to our work and others cited above is the reliance on examining hedgehogs that are rescued or found dead. Disease prevalence in rescued hedgehogs might be higher compared to the overall population due to an overrepresentation of weak and diseased hedgehogs in rehabilitation. It was beyond the scope of our study to determine the prevalence of periodontal disease in the overall Western European hedgehog population of northwestern Germany.

Different authors have suggested that a soft diet or the thereby triggered reduced chewing time, and different nutritional components, like high sugar and low mineral contents, can have a negative influence on the dental apparatus (Zuhrt 1958; Sainsbury et al. 1996; Bexton 2016; Stepaniuk 2019). Rautio et al. (2016) found that 92% of hedgehogs in a Finnish city whose stomach contents were examined, had eaten anthropogenic food, mainly fish, milk, and unidentified meat. Hubert et al. (2011) found the pet food abundance to be 2.6 times higher in urban compared to rural areas. As most hedgehogs in this study came from an urban environment, it can be assumed that they had eaten anthropogenic food to some extent.

According to the literature, periodontal disease can be found with or without the presence of calculus (Stepaniuk 2019). In this study, gingivitis without calculus was only found in two young hedgehogs currently in tooth change. Therefore, it is likely that gingivitis was present due to this physiological process. Calculus was the most common pathology which is in accordance with the literature (Poduschka and Poduschka 1986; Koegel 2009). These findings suggest that the disease complex starts to become visible with the building of calculus in Western European hedgehogs. All individuals who showed calculus and no other pathologies had a calculus index of 1. For the periodontal index and the overall examination index, high scores were more common than medium ones. Therefore, it is likely that once the buildup of calculus advances, gingivitis and periodontal recession appear and progress quickly. Calculus alone does not seem to influence the affected individuals significantly, but the following inflammatory reaction must be assumed to be painful with potential local or systemic effects on other tissues (Botting and Botting 2000; Niemiec 2008).

The indices developed for this study are not yet validated but drawn from standard clinical observations. Limitations include the fact that associated pathologies like tooth-root abscesses, which can influence the prognosis, are not represented and in individual cases, an index can be influenced by a physiological process like tooth change. Whilst we acknowledge these limitations we believe that the final assessment comprising the sum of four individual indices equally weighted provides a realistic representation of the disease complex’ characteristics and offers an easy-to-use, comparable method to describe dental and periodontal pathologies in hedgehogs under anaesthesia.

The finding that dental/periodontal disease is more common in adults compared to juvenile individuals is in accordance with the literature on hedgehogs and other species (Chaprazov et al. 2014; Bexton 2016; Stepaniuk 2019). Currently, invasive methods are needed to determine the exact age of hedgehogs (Rasmussen et al. 2023). Therefore, a more detailed investigation on the influence of increasing age on dental/periodontal pathologies was not possible. As 89.5% of hedgehogs past their first calendar year and even 14% of juveniles in this study showed pathologies, it is unlikely that only elderly individuals develop dental/periodontal disease.

A higher body weight was positively associated with the presence of dental/periodontal disease in both age groups in this study. Amongst hedgehogs within their first calendar year, this is likely due to the low chance of dental/periodontal disease while having their primary dentition and the rapidly increasing body weight within their first months of life. In hedgehogs past their first calendar year, this is surprising as from the literature one could expect that the disease affects feed intake and leads to emaciation. The results might suggest that heavier hedgehogs have eaten more high-energy and soft (likely anthropogenic) food sources. Alternatively, the older and therefore heavier hedgehogs in this age group might be responsible for the statistical significance. As Western European hedgehogs only reach sexual maturity in their second year of life, there are still growing individuals within this age group (Berger et al. 2023).

Further investigation of the impact of dental and periodontal disease on weight development and feeding behaviour, blood analyses and post-mortem examinations (potential systemic effects), and post-release monitoring (long-term disease development and survival, impact on reproductive behaviour) would be useful. Future research should also concentrate on disease prevalence among otherwise healthy hedgehogs in the wild, and the effect of various diets, particularly anthropogenic diets, on hedgehogs’ dental and overall health.

In conclusion, this study shows that dental/periodontal disease is a common finding among hedgehogs admitted into rehabilitation in northwestern Germany, with calculus found to be the most common pathology. Dental examination under general anaesthesia followed by appropriate treatment can be recommended for all individuals above 250 g entering rehabilitation. Further research is needed to understand the significance of the disease complex to the individual and the declining overall Western European hedgehog population.

## Electronic Supplementary Material

Below is the link to the electronic supplementary material.

Online Resource 1: Examination protocol including explanations and a visual presentation of the dental indices.

## Data Availability

The data collected are available as open data via the University of Edinburgh online data repository: https://doi.org/10.7488/ds/7983.

## References

[CR1] Berger A, Geiger M, Taucher AL (2023) Sustainable protection of hedgehog populations in urban and rural habitats. In: Voigt CC (ed) Evidenzbasiertes Wildtiermanagement. Springer Spektrum, Berlin, pp 103–126

[CR2] Bexton S (2016) Hedgehogs. In: Mullineaux E, Keeble E (eds) BSAVA Manual of Wildlife Casualties, 2nd edn. British Small Animal Veterinary Association, Quedgeley, pp 117–136

[CR3] Botting RM, Botting JH (2000) Pathogenesis and mechanisms of inflammation and pain. Clin Drug Invest 19:1–7. 10.2165/00044011-200019002-00001

[CR4] Boulton CH (1985) Equine nasal cavity and paranasal sinus disease: A review of 85 cases. J Equine Vet Sci 5(5):268–275. 10.1016/S0737-0806(85)80062-9

[CR5] Chaprazov T, Dimitrov R, Stamatova Yovcheva K, Uzunova K (2014) Oral and dental disorders in pet hedgehogs. Turk J Vet Anim Sci 38(1):1–6. 10.3906/vet-1302-46

[CR6] Doepke C (2002) Kasuistische Auswertung der Untersuchungen von Igeln (*Erinaceus europaeus*) im Einsendungsmaterial des Instituts für Pathologie von 1980 bis 2001. Dissertation, Tierärztliche Hochschule Hannover

[CR7] Heatley JJ (2009) Hedgehogs. In: Mitchell MA, Tully TN (eds) Manual of Exotic Pet Practise. Saunders Elsevier, St. Louis, pp 433–455

[CR8] Hubert P, Julliard R, Biagianti S, Poulle ML (2011) Ecological factors driving the higher hedgehog (*Erinaceus europeaus*) density in an urban area compared to the adjacent rural area. Landsc Urban Plan 103(1):34–43. 10.1016/j.landurbplan.2011.05.010

[CR9] Hungerford LL, Mitchell MA, Nixon CM, Esker TE, Sullivan JB, Koerkenmeier R, Marretta SM (1999) Periodontal and dental lesions in raccoons from a farming and a recreational area in Illinois. J Wildl Dis 35(4):728–734. 10.7589/0090-3558-35.4.728 10574532 10.7589/0090-3558-35.4.728

[CR10] Koegel B (2009) Untersuchungen zu Igelpfleglingen ausgewählter deutscher Igelstationen und Erfolge der Therapie aus den Jahren 1984 bis 2006. Dissertation, Tierärztliche Hochschule Hannover

[CR11] Malmsten A, Dalin AM, Pettersson A (2015) Caries, Periodontal Disease, Supernumerary Teeth and Other Dental Disorders in Swedish Wild Boar (*Sus scrofa*). J Comp Pathol 153(1):50–57. 10.1016/j.jcpa.2015.04.003 25979683 10.1016/j.jcpa.2015.04.003

[CR12] Molina-Lopez RA, Obón E, Darwich L (2024) Morbidity and prognostic factors associated with wild hedgehogs admitted to a wildlife rehabilitation center in Catalonia (NE Spain) from 1995 to 2020. 10.3390/ani14040556. Animals 14,556 10.3390/ani14040556 PMC10886239 38396523

[CR13] Niemiec BA (2008) Periodontal disease. Top Companion Anim Med 23(2):72–80. 10.1053/j.tcam.2008.02.003 18482707 10.1053/j.tcam.2008.02.003

[CR14] Poduschka W, Poduschka C (1986) Zahnstein, Zahnfleischerkrankungen und Zahnanomalien bei Erinaceinen (*Mammalia Insektivora*). Z für angewandte Zool 73:231–243

[CR15] Rasmussen SL, Berg TB, Martens HJ, Jones OR (2023) Anyone can get old – all you have to do is live long enough: Understanding mortality and life expectancy in European hedgehogs (*Erinaceus europaeus*), Animals 13. 626. 10.3390/ani13040626 36830413 10.3390/ani13040626 PMC9951656

[CR16] Rautio A, Isomursu M, Valtonen A, Hirvelä-Koski V, Kunnasranta M (2016) Mortality, disease and diet of European hedgehogs (*Erinaceus europaeus*) in an urban environment in Finland. Mammal Res 61:161–169. 10.1007/s13364-015-0256-7

[CR17] Sainsbury AW, Cunningham AA, Morris PA, Kirkwood JK, Macgregor SK (1996) Health and welfare of rehabilitated juvenile hedgehogs (*Erinaceus europaeus*) before and after release into the wild. Vet Rec 138:61–65. 10.1136/vr.138.3.61 8629331 10.1136/vr.138.3.61

[CR18] Simone-Freilicher E, Hoefer H (2004) Hedgehog care and husbandry. Vet Clin North Am Exot Anim Pract 7(2):257–267. 10.1016/j.cvex.2004.01.004 15145389 10.1016/j.cvex.2004.01.004

[CR19] Stepaniuk K (2019) Periodontology. In: Lobprise HB, Dodd JR (eds) Wigg’s Veterinary Dentistry: Principles and Practice, 2nd edn. Wiley Blackwell, pp 81–108

[CR20] Verstraete F (2003) Advances in diagnosis and treatment of small exotic mammal dental disease. Sem Avian Exot Pet Med 12(1):37–48. 10.1053/saep.2003.127877

[CR21] Zuhrt R (1958) Zahnfleischerkrankungen beim Igel als Todesursache. Der Zoologische Garten 24:74–80

