# Supplementary material for: Prevalence and characteristics of dental and periodontal disease in Western European hedgehogs (Erinaceus europaeus) admitted into an animal shelter in northwestern Germany
Source: Vet Res Commun. 2026 May 22;50(4):340. doi: 10.1007/s11259-026-11287-0 (PMC13197348; doi:10.1007/s11259-026-11287-0)
Supplement: Supplementary file 1 — Online Resource 1: Examination protocol including explanations and a visual presentation of the dental indices. [file 11259_2026_11287_MOESM1_ESM.pdf]

Title:

Prevalence and characteristics of dental and periodontal disease in Western European hedgehogs (*Erinaceus europaeus*) admitted into an animal shelter in northwestern Germany

Veterinary Research Communications

Authors:

Ines Stegmaier-Länge[1,2]

Gidona Goodman[1]

Lian Thomas[1,3] ORCID: 0000-0001-8447-1210

Corresponding author: lian.thomas@ed.ac.uk

Affiliations:

[1]Royal (Dick) School of Veterinary Studies, University of Edinburgh, Edinburgh, United Kingdom

[2]Bremer Tierschutzverein e.V., Bremen, Germany

[3]International Livestock Research Institute, Nairobi, Kenya

Veterinary examination protocol for hedgehogs

Hedgehog number: ______ Date: ______

State: Alive / Dead

Sex: Male / Female

Oral examination:

Dental and/or periodontal disease: No / Yes

Dental indices:

Gingival index: ____

Calculus index: ____

Periodontal index: ____

Mobility index: ____

Other oral abnormalities: No / Yes: ____________________

Dental treatment necessary: No / Yes / Yes but euthanasia

Other abnormalities during full body examination:

Respiration: No / Yes: ____________________

Skin/quills: No / Yes: ____________________

Nose: No / Yes: ____________________

Eyes: No / Yes: ____________________

Ears: No / Yes: ____________________

Palpation of musculoskeletal system: No / Yes: ____________________

Anus und genital tract: No / Yes: ____________________

Palpation of abdomen: No / Yes: ____________________

Body condition: Good / Reduced / Adipose

Weight: ____ g

Body length: ____ cm

Age: Juvenile / > First calendar year

Explanations:

Hedgehog number: Assigned upon arrival in consecutive order of arrival.

State:1 Alive 2 Dead

Sex (1 Male 2 Female): The sex in hedgehogs is easily identifiable under general anaesthesia as the penis sheath is located centred on the abdomen whereas the vulva is located close to the anus.

Oral examination:

Dental and/or periodontal disease (0 no 1 yes): Choose yes if any abnormalities on the dental or periodontal apparatus are present.

Dental indices: see following page

Dental assessment in hedgehogs

Dental formula of a European hedgehog:

Adult dentition: 36 teeth

I	C	P	M
3	1	3	3
2	1	2	3

Primary dentition: 22 teeth

Calculus index:

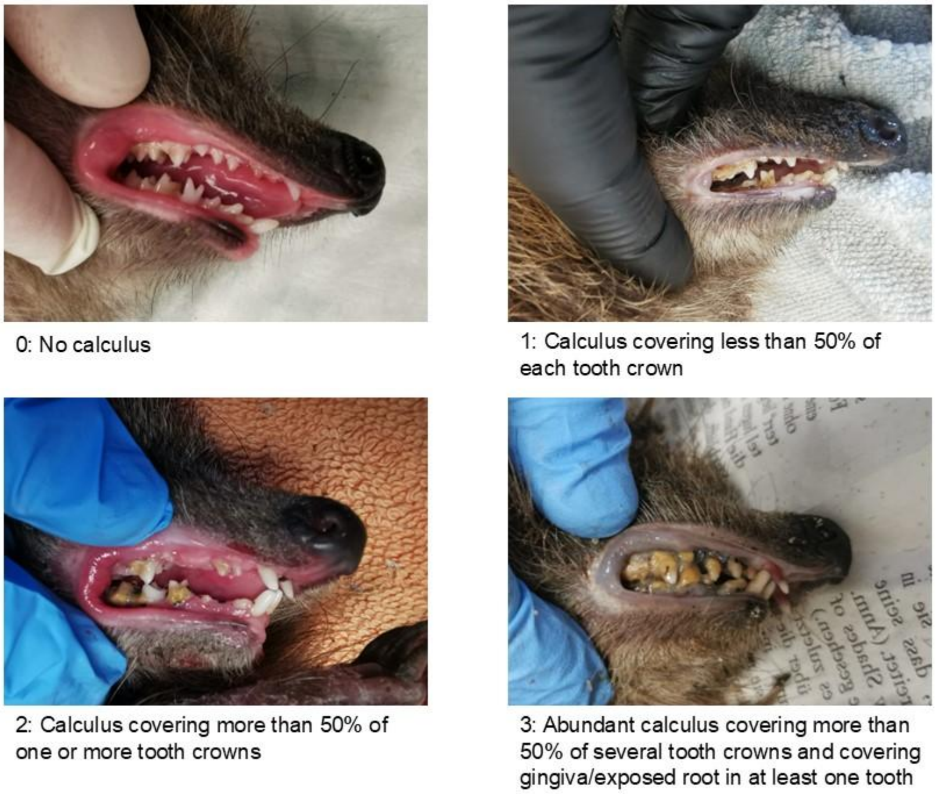

Figure 1 Calculus index (photos: Ines Stegmaier-Länge)

Gingival index:

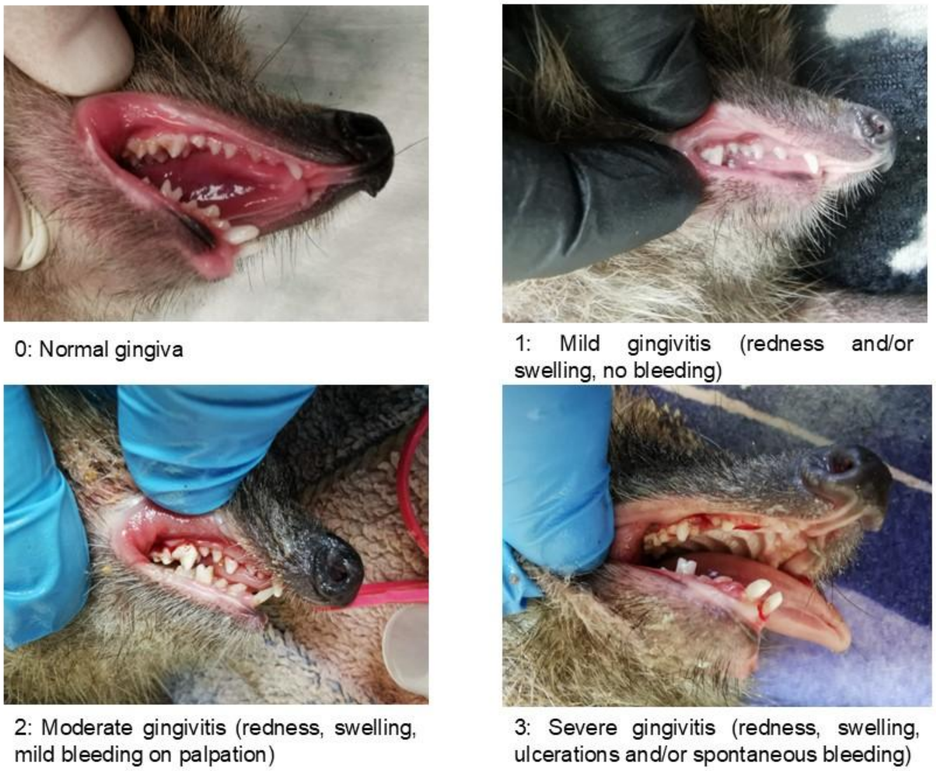

Figure 2 Gingival index (photos: Ines Stegmaier-Länge)

Periodontal index:

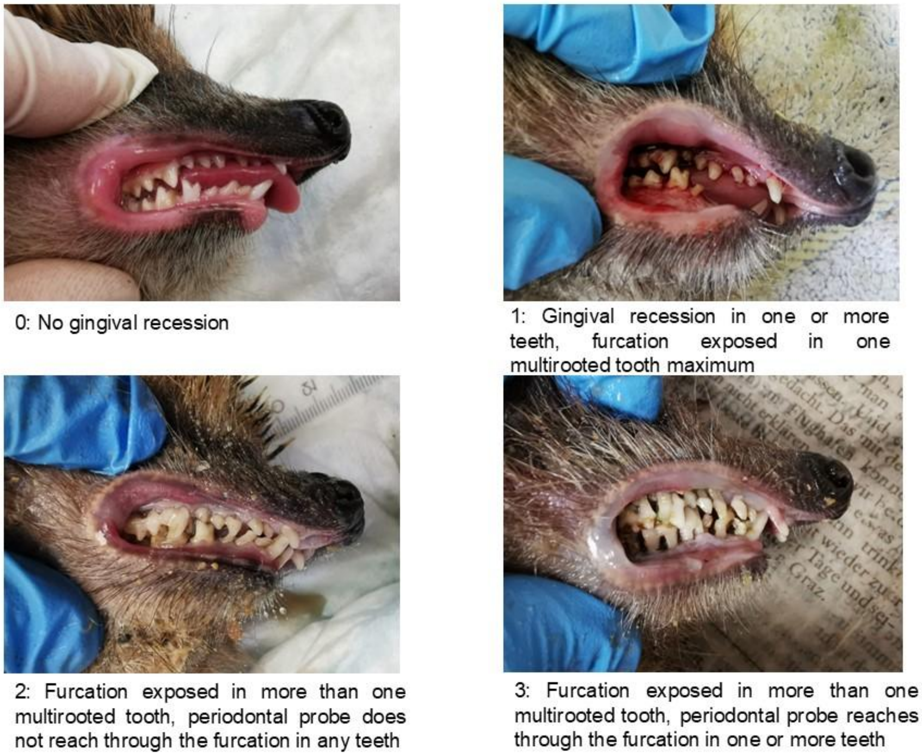

Figure 3 Periodontal index (photos: Ines Stegmaier-Länge)

Mobility index:

0: No noticeable mobility

1: Mildly increased movement of one or more teeth which stay stable within the alveolus

2: Up to four teeth loose and likely to fall out soon, no or mildly increased movement in other teeth

3: More than four teeth loose or likely to fall out soon, no or mildly increased movement in other teeth

Other oral abnormalities (0 no 1 yes): Note anything abnormal that is not covered by the dental indices. This might include abscesses or broken teeth.

Respiration: The normal respiratory rate in a hedgehog is 20 to 50 per minute. Note anything unusual like an abnormal respiratory rate or breathing noise.

Skin/quills: Note missing or broken quills, skin alterations like baldness or scaly skin and check full body for wounds or swelling.

Nose: Note anything abnormal like unusual amount of nasal discharge from one or both nares (a small amount of clear discharge from both nares is normal), check for fly eggs or maggots.

Eyes: Note anything abnormal like ocular discharge or swelling on one or both sides.

Ears: Note any signs of otitis like unusual high amount of dirt, redness or puss, check for fly eggs or maggots.

Palpation of musculoskeletal system: Palpate all limbs for signs of fractures like swelling or crepitation.

Anus und genital tract: Check for abnormal discharge and fly eggs or maggots.

Palpation of abdomen: Palpate abdomen from directly behind the ribs to the back, note signs of abnormal mass growth or unusual firmness.

Body condition: Body condition is good if the body is shaped like a pear when looking from the top (with the rump being wider than the thorax region) and there is no neck visible (no so-called "hunger kink"). An adipose animal has fat deposits palpable all over the body.

Weight: Place scales on an even surface and note weight in grams.

Body length: Measure body length in centimetres when fully expanded from the tip of the nose to the rump.

Age: Differentiation between juveniles in their first calendar year and/or with juvenile dentition and adults (> first calendar year with full adult dentition) according to anamnesis (time of year) and morphological criteria (body weight, body condition, body length, dentition).
